# Supplementary material for: Advanced clustering and transfer learning based approach for rice leaf disease segmentation and classification
Source: PeerJ Comput Sci. 2025 Jul 28;11:e3018. doi: 10.7717/peerj-cs.3018 (PMC12453724; doi:10.7717/peerj-cs.3018)
Supplement: Supplemental Information 1 [file peerj-cs-11-3018-s001.docx]

Table S 1: Comparison of Methods for Rice Leaf Disease Detection

| **Ref** | **Method** | **Dataset** | **Accuracy (%)** | **Limitation** |
| --- | --- | --- | --- | --- |
| Mekha and  Teeyasuksaet  (2021) | Random Forest | Rice leaf diseases dataset from UCI | 69.4 | Insufficient classification accuracy performance. |
| Thepade et al.  (2022) | Otsu thresholding and Thepade sorted block truncation coding | International Rice Research Institute (IRRI) | 85.9 | Additional enhancements needed for disease classification. |
| Haridasan  et al. (2023) | CNN | Self-generated database | 91.4 | Classification accuracy may improve with preprocessing. |
| Jiang et al.  (2020) | CNN and SVM | Self-generated database | 96.8 | Works well only with high-quality images. |
| Azim et al.  (2021) | Extreme Gradient Boosting | Rice leaf diseases dataset from UCI | 86.5 | Small dataset size. |
| Liang et al.  (2019) | DCNN | Images from the Institute of Plant Protection | 95.8 | Focuses on only one rice disease. |
| Lu et al.  (2017) | DCNN | Self-generated database | 95.4 | Time-consuming due to deep learning architecture complexity. |
| Krishnamoorthy  et al. (2021) | InceptionResNetV2 | Self-generated database | 95.6 | Manual hyperparameter selection; optimization algorithms could improve performance. |
| Wang et al.  (2022) | Attention-based Neural Network with Bayesian Optimization (ADSNN-BO) | Manually curated rice leaf disease dataset (2370 images) | 94.6 | Limited dataset size, potential generalization issues, and need for validation on diverse datasets. |
| Shanmugam  et al. (2023) | SEWA-SPBO optimized Deep Maxout Network with BHEFC Segmentation | Self-captured rice leaf images using Sony RX 100 IV | 93.9 | Small dataset size, controlled lighting dependency, and limited field applicability. |
