# Supplementary figures and images for: Advanced clustering and transfer learning based approach for rice leaf disease segmentation and classification
Source: PeerJ Comput Sci. 2025 Jul 28;11:e3018. doi: 10.7717/peerj-cs.3018 (PMC12453724; doi:10.7717/peerj-cs.3018)

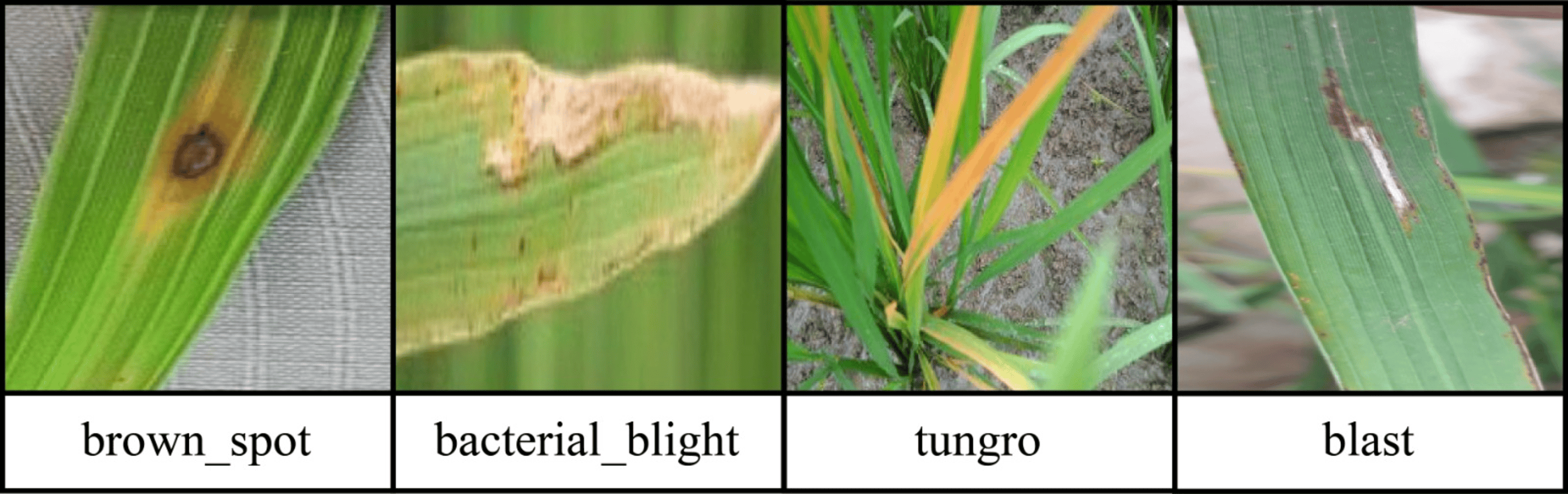

Supplement: Supplemental Information 2 [file peerj-cs-11-3018-s002.png]

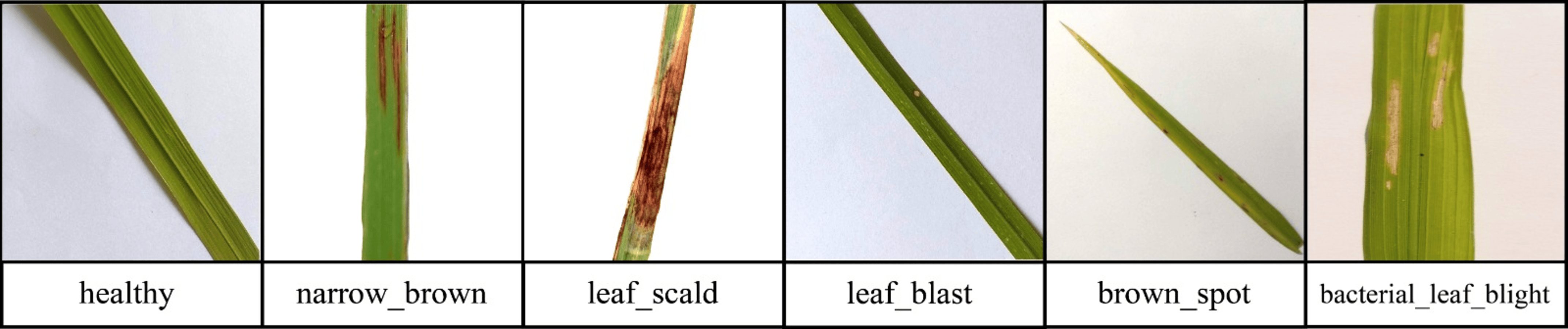

Supplement: Supplemental Information 3 [file peerj-cs-11-3018-s003.png]

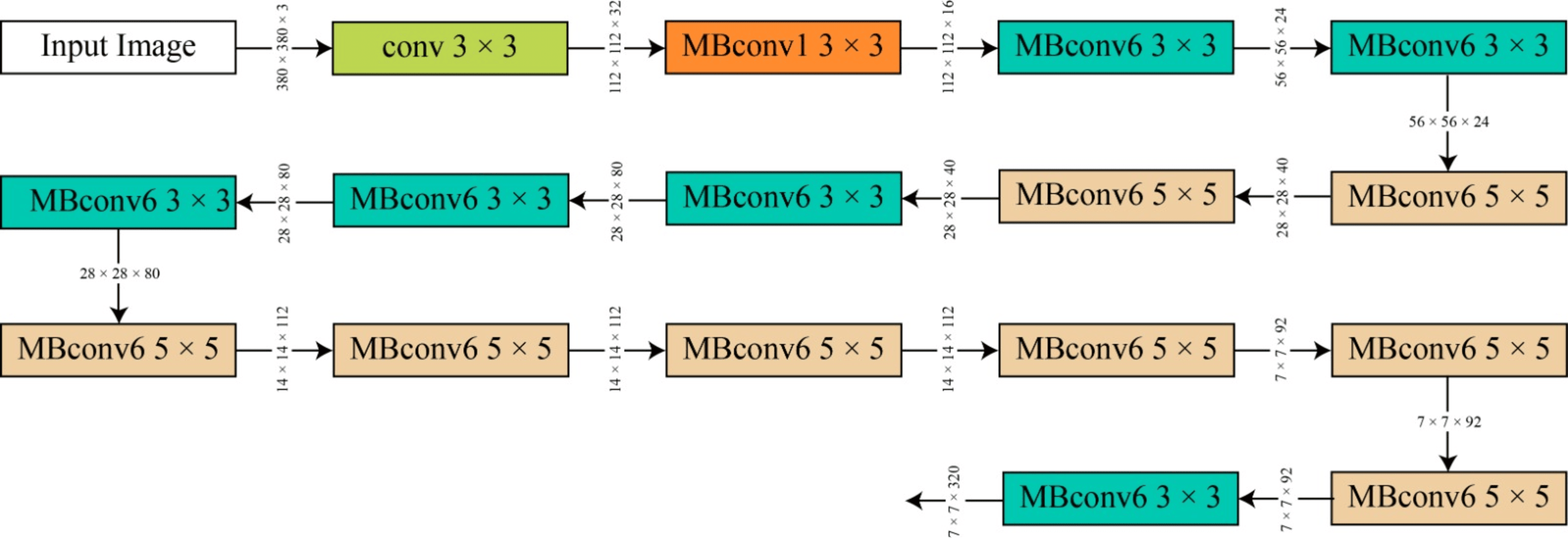

Supplement: Supplemental Information 4 [file peerj-cs-11-3018-s004.png]

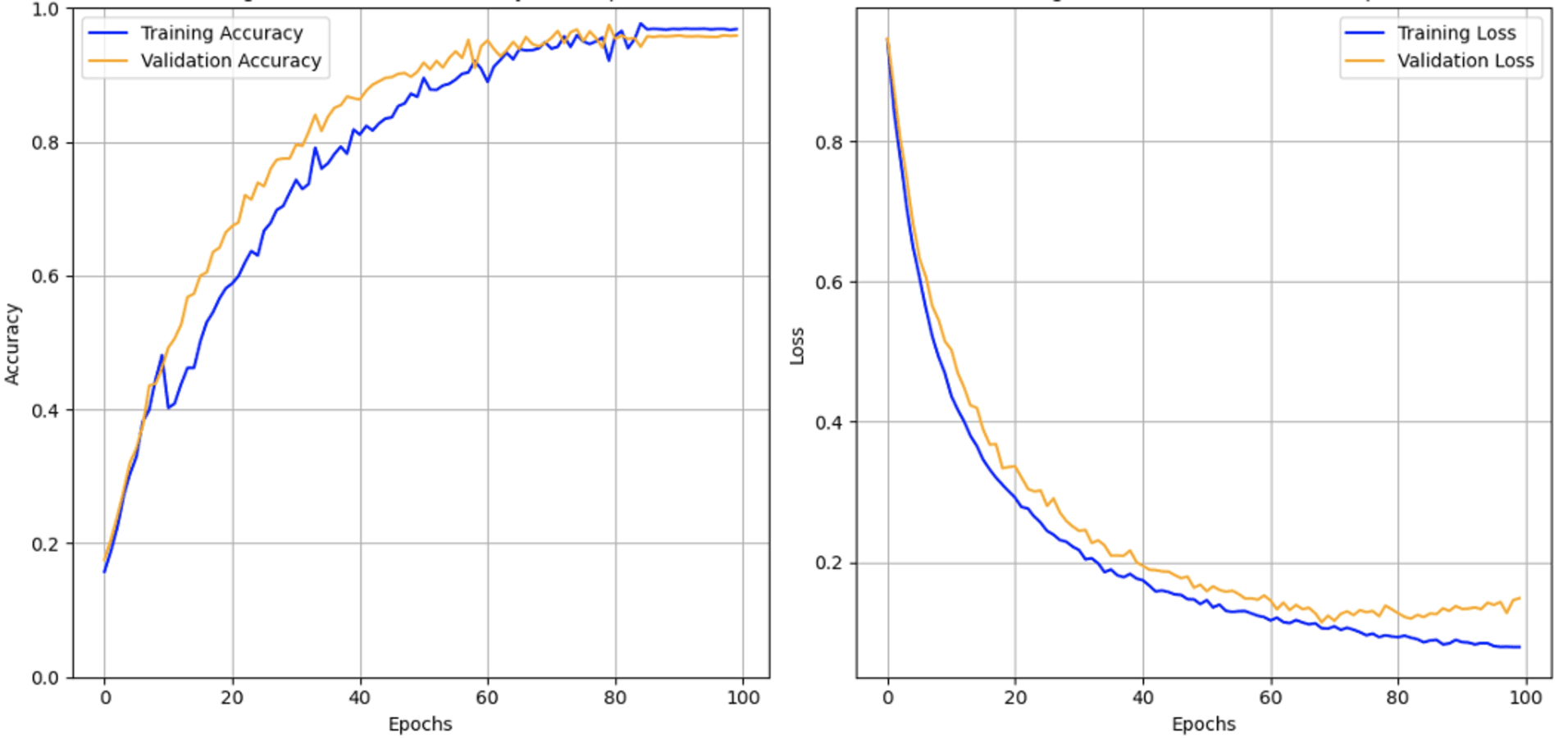

Supplement: Supplemental Information 5 [file peerj-cs-11-3018-s005.png]

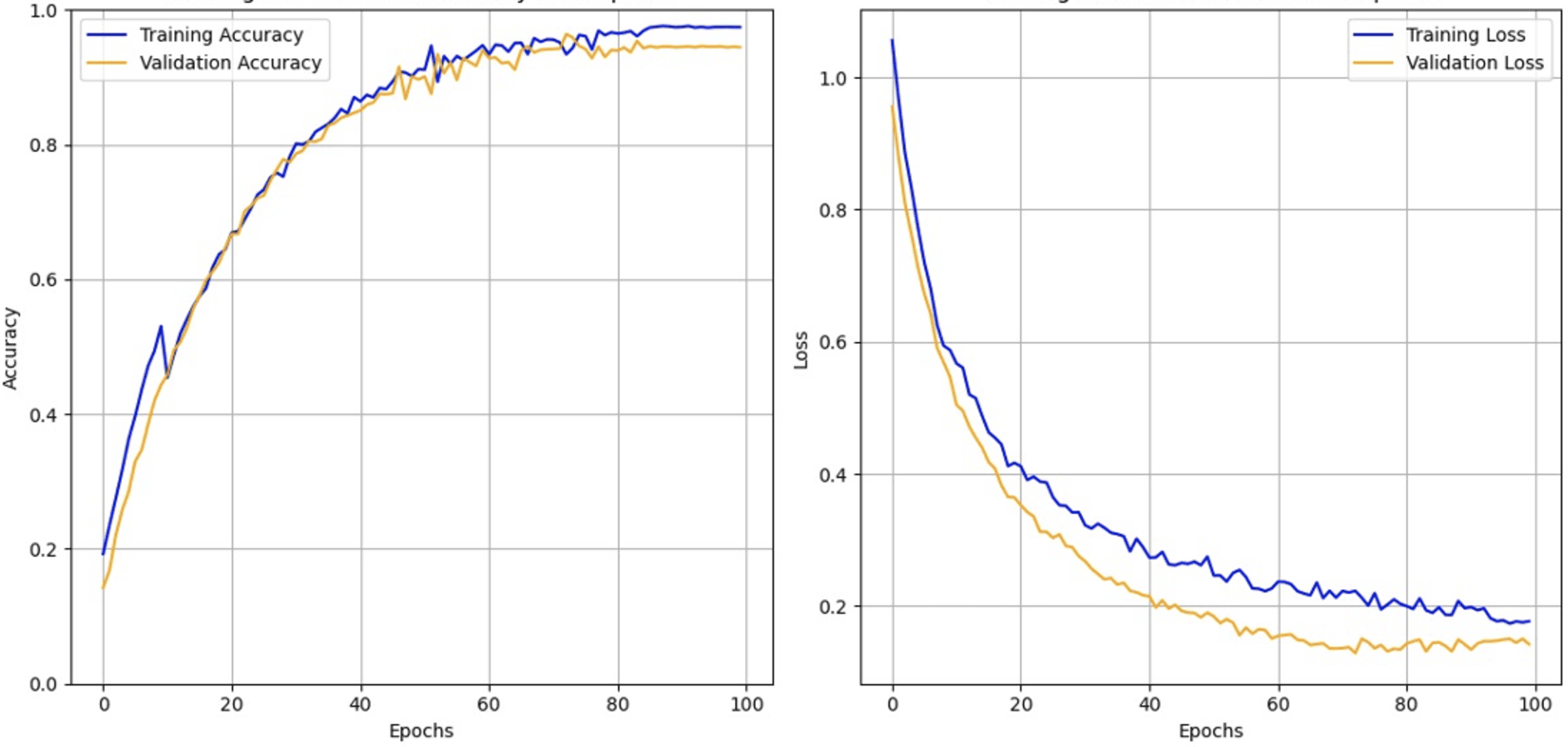

Supplement: Supplemental Information 6 [file peerj-cs-11-3018-s006.png]

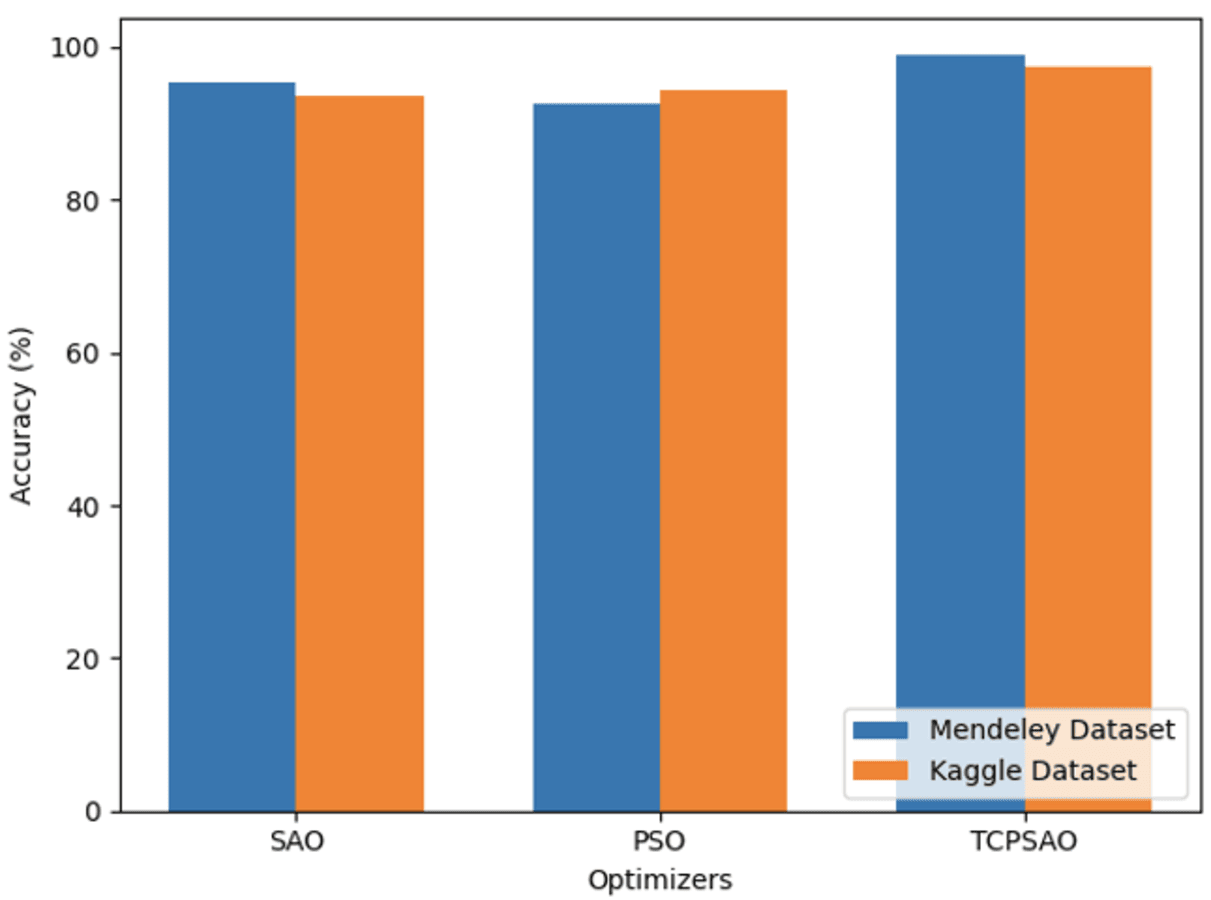

Supplement: Supplemental Information 7 [file peerj-cs-11-3018-s007.png]
